# Automatic and Reproducible Positioning of Phase-Contrast MRI for the Quantification of Global Cerebral Blood Flow

**DOI:** 10.1371/journal.pone.0095721

**Published:** 2014-05-02

**Authors:** Peiying Liu, Hanzhang Lu, Francesca M. Filbey, Amy E. Pinkham, Carrie J. McAdams, Bryon Adinoff, Vamsi Daliparthi, Yan Cao

**Affiliations:** 1 Advanced Imaging Research Center, University of Texas Southwestern Medical Center, Dallas, Texas, United States of America; 2 Department of Psychiatry, University of Texas Southwestern Medical Center, Dallas, Texas, United States of America; 3 Center for Brain Health, School of Behavioral and Brain Science, University of Texas at Dallas, Dallas, Texas, United States of America; 4 Department of Psychology, Southern Methodist University, Dallas, Texas, United States of America; 5 VA North Texas Health Care System, Dallas, Texas, United States of America; 6 Department of Mathematical Sciences, University of Texas at Dallas, Richardson, Texas, United States of America; University of Medicine & Dentistry of NJ - New Jersey Medical School, United States of America

## Abstract

Phase-Contrast MRI (PC-MRI) is a noninvasive technique to measure blood flow. In particular, global but highly quantitative cerebral blood flow (CBF) measurement using PC-MRI complements several other CBF mapping methods such as arterial spin labeling and dynamic susceptibility contrast MRI by providing a calibration factor. The ability to estimate blood supply in physiological units also lays a foundation for assessment of brain metabolic rate. However, a major obstacle before wider applications of this method is that the slice positioning of the scan, ideally placed perpendicular to the feeding arteries, requires considerable expertise and can present a burden to the operator. In the present work, we proposed that the majority of PC-MRI scans can be positioned using an automatic algorithm, leaving only a small fraction of arteries requiring manual positioning. We implemented and evaluated an algorithm for this purpose based on feature extraction of a survey angiogram, which is of minimal operator dependence. In a comparative test-retest study with 7 subjects, the blood flow measurement using this algorithm showed an inter-session coefficient of variation (CoV) of 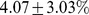. The Bland-Altman method showed that the automatic method differs from the manual method by between 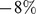 and 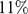, for 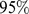 of the CBF measurements. This is comparable to the variance in CBF measurement using manually-positioned PC MRI alone. In a further application of this algorithm to 157 consecutive subjects from typical clinical cohorts, the algorithm provided successful positioning in 89.7% of the arteries. In 79.6% of the subjects, all four arteries could be planned using the algorithm. Chi-square tests of independence showed that the success rate was not dependent on the age or gender, but the patients showed a trend of lower success rate (p = 0.14) compared to healthy controls. In conclusion, this automatic positioning algorithm could improve the application of PC-MRI in CBF quantification.

## Introduction

Phase-Contrast MRI (PC-MRI) is a noninvasive technique to visualize blood vessels and measure blood flow velocity. It utilizes the phase of an image to encode the velocity of flowing spins [1] and has been validated for angiogram and quantitative flow measurements [2]–[4].

One of the major utilities of PC-MRI is to quantify whole-brain cerebral blood flow (CBF), by measuring and combining flow flux at the main feeding arteries of the brain [5]–[10], e.g. left/right internal carotid arteries (ICA) and left/right vertebral arteries (VA). Comparing to other neuroimaging methods for the evaluation of CBF, the most significant advantages of PC-MRI are its simplicity and accuracy in absolute CBF quantification as well as the relatively short scan duration. As a result, although PC-MRI cannot provide spatial information of blood flow distribution, it has increasingly been used in three major areas of clinic and research applications. First, the knowledge of CBF, combined with quantitative evaluation of cerebral arterial and venous oxygenation, provides direct assessment of a highly desirable index, cerebral metabolic rate of oxygen (CMRO2) [10]–[15]. Second, it can be used for the calibration of other qualitative CBF methods such as Dynamic Susceptibility Contrast (DSC) and Arterial Spin Labeling (ASL) MRI, in which quantification is traditionally confounded by factors such as arterial input function or labeling efficiency [5], [6]. Third, with the recent development of simultaneous Positron Emission Tomography (PET) and MRI system, the PET CBF measurement procedures can be simplified considerably by using PC-MRI to avoid the need of sampling arterial blood during PET scanning. That is, one can acquire a relative PET CBF map without arterial sampling and then utilizes global CBF measured from PC-MRI to obtain a PET-MR “hybrid” CBF map that is both quantitative and spatially resolved.

It is therefore important to establish a reliable protocol of PC-MRI for the accurate quantification of whole brain CBF at the brain’s feeding arteries. The PC-MRI scans are usually positioned based on a 3-D time-of-flight (TOF) angiogram of the neck region. At present, the standard practice is that the operator inspects the 2-D maximum-intensity-projection (MIP) of the 3-D images, identifies the ICA and VA vessels, and selects a plane that is perpendicular to target vessel at the entry point of the skull. However, given large variability in arterial anatomy across individuals, the slice positioning of the PC-MRI scans requires considerable training and expertise for the operator and contributes to measurement noise. Perhaps more importantly, these requirements place a significant burden and stress on the MR technologist, which may result in resistance and obstacles when applying this technique in a broader scope of applications.

In the present work, we aimed to alleviate this practical obstacle by developing an algorithm that is capable of automatically planning the PC-MRI scans in the majority of arteries, leaving only a small fraction that requires manual positioning. The performance of this automatic positioning algorithm was first evaluated by a test-retest study, in which the reproducibility was evaluated by comparing the measurements of whole brain CBF between repeated sessions. Accuracy was evaluated by comparing results using the automatic algorithm to those based on manual positioning by a highly experienced operator. In a second, larger scale study, we demonstrated the practicality of this automatic positioning algorithm in 157 subjects over a wide age range, which provided more accurate information on the success rate, reasons for failure, and dependence of algorithm performance on age and gender of the subjects.

## Methods

### The Automatic Positioning Algorithm

**Figure 1:**
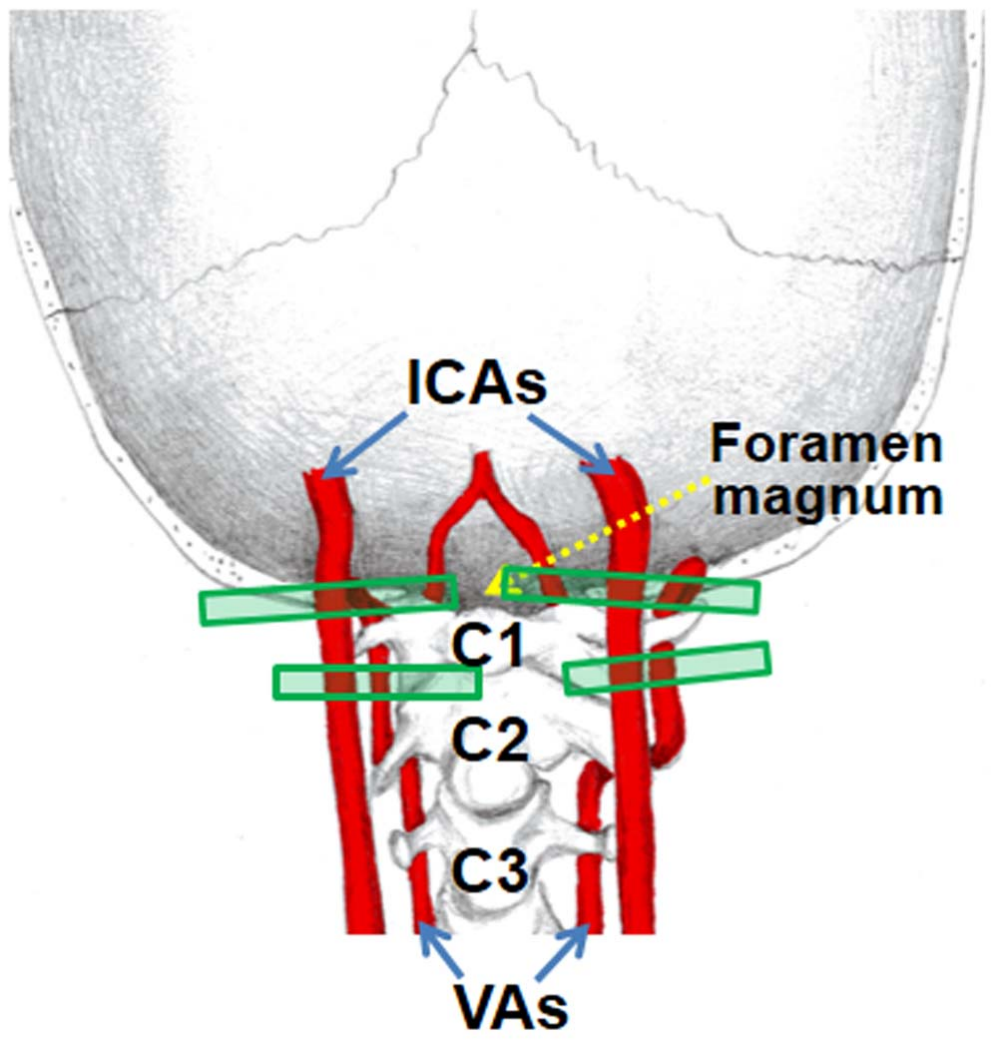
An ideal case of scan planning. The ICA scans were placed at the level of foramen magnum, perpendicular to the trajectory of the vessels. The VA scans were placed in the middle of the two turns at the level of cervical vertebra 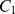 and 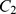, perpendicular to the trajectory of the vessels.

The general goal of PC-MRI for global CBF determination is to measure the flow flux at the location where the feeding arteries enter the brain, referred to as foramen magnum in anatomy literature. Since the brain is supplied exclusively by four arteries, left/right ICA and left/right VA, we will perform four PC-MRI scans, with each targeting one specific artery. The automatic positioning algorithm includes two major steps: 1) identification of the four brain feeding arteries, left/right ICA and left/right VA, from the angiogram, and 2) determination of the optimal positioning for PC-MRI scans (one scan for each artery) based on the geometric properties of the arteries. Figure 1 illustrates an ideal case of scan planning following automatic positioning. The ICA scans were placed at the level of foramen magnum, perpendicular to the trajectory of the vessels. The VAs, on the other hand, are known to travel medially and posteriorly at this location [16], making the trajectory of the vessel unpredictable. We therefore aimed to place the VA scan planes at a slightly distal position, between the two turns at the level of cervical vertebra 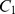 and 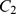 on its trajectory, where the vessels are straighter. In addition, algorithmic considerations were made such that the computation is sufficiently fast to be used in situ during a scan session.

#### Identification of brain feeding arteries

**Figure 2:**
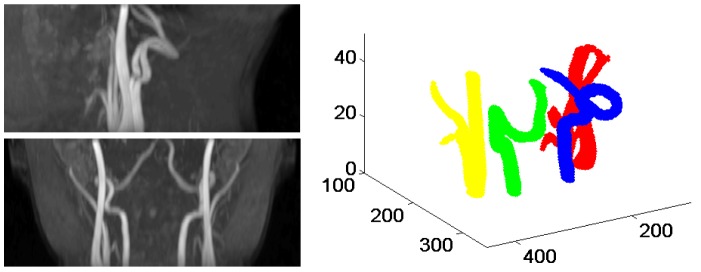
Identification of the brain feeding arteries from the angiogram. Top left two panels show the maximum intensity projection (MIP) of the arteries in two planes. Top right panel shows the 3D segmentation results of the carotid arteries and vertebral arteries.

We first used the Otsu’s method [17] to define voxels inside the body 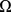 from those in the air. The Otsu’s method chooses the threshold to minimize the intraclass variance of the object and background pixels. The object region 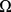 included both the arteries and the surrounding tissues. Then we used the mean 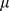 and the standard deviation 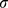 of the object region to define the threshold for the arteries. Let
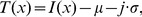
(1)where 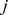 is an integer. The artery region consisted the pixels 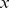 such that 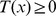. Since intensities of the arteries are not homogeneous, we started with a high value of 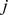 and reduced it gradually to find the optimal value. We used the length of the arteries in 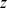 (F–H) direction to determine if the segmentation result was satisfactory. We picked an integer 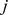 such that the length of the arteries in 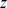 direction was at least 60% of the scan range in 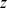 direction. This was to ensure that we extract the portion of the vertebral arteries between the two turns at the levels of the cervical vertebra 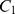 and 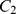. See Figure 2 for an example. The value of j was determined on a subject-by-subject basis. Note that j should be chosen such that it is high enough to ensure that static tissue is excluded while it is low enough to include the arterial vessels. A search range of 5 to 3 in descending order was used in our experiments. The upper bound value of 5 was chosen because few tissue voxels would exceed mean plus 5 times standard deviation. At this threshold, if all four arteries are detected successfully (i.e. length greater than or equal to 60% of the FOV along 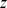 direction), then the segmentation step is completed. If not, the threshold is reduced (by 1) and the lengths of the arteries are evaluated again. This process is repeated until a minimum of 60% of each artery was depicted or the 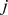 value has reached its lower bound value, 3. We stopped at a threshold at 3 because, if we lower the threshold to be less than 3, then static tissue clusters would appear, at which point we could not distinguish an artery from a tissue cluster. Therefore, we decided empirically that, if at threshold of 3 we still could not find an artery, then that artery was too noisy (low intensity) to be identified.

**Figure 3:**
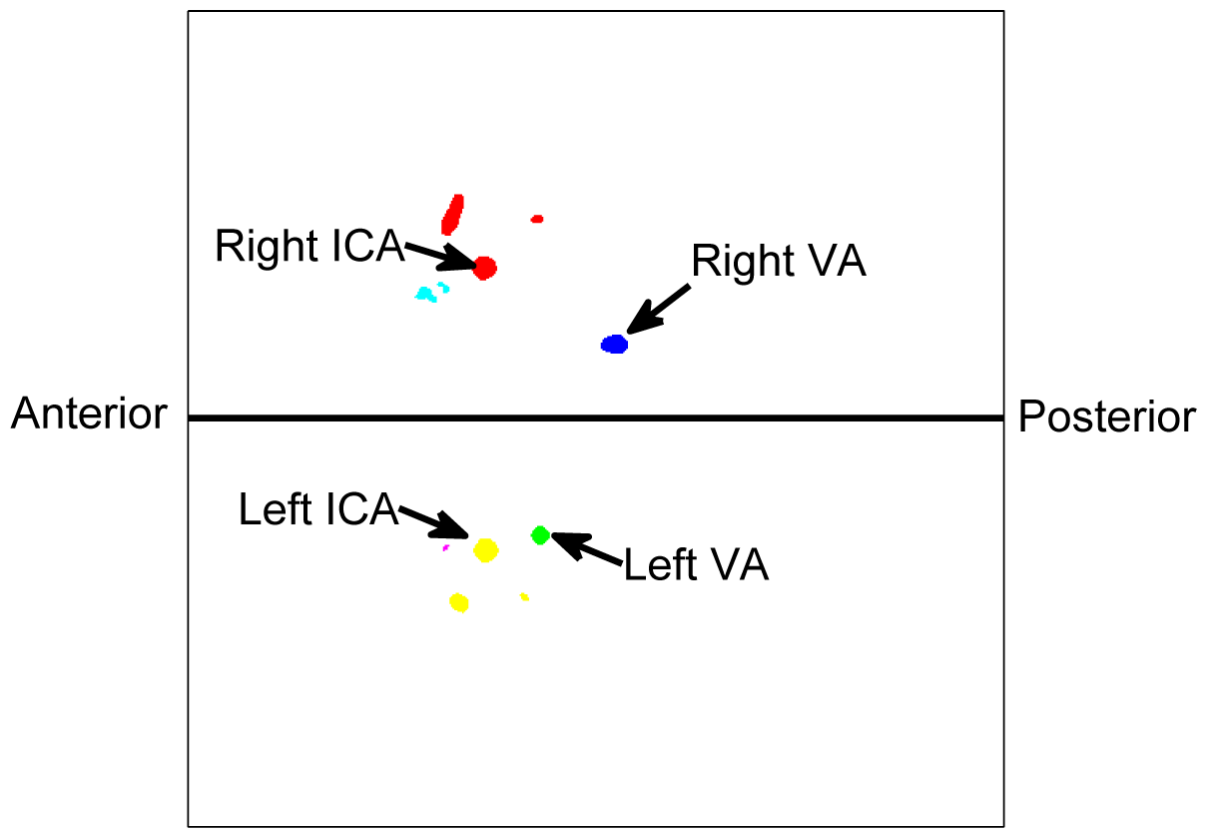
Detection of left and right VAs, left and right ICAs using the middle slice of the thresholded image volume. The cross sections from the same object are represented by the same color.

After thesholding, most arteries were segmented out as connected objects. We were interested in detecting the left/right ICA and left/right VA. These are the major brain feeding arteries and were always placed in the center area of the image volume. We used this information to identify them in the image volume. We first identified the eight largest connected objects as the candidates. We then used the middle slice in 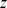 direction to find the four arteries we were interested in (See Figure 3). In the middle slice, each artery branch was represented by the cross section. Note that one connected object may have multiple cross sections because of the existence of branches. We represented each cross section by its centers. Among the eight connected objects, the VAs were the furthest in the posterior direction and had no branches. In addition, left VA was located in the left half of the image and right VA was located in the right half of the image. After detecting the VAs, the carotid arteries were identified as the two largest remaining objects with one in the left half and one in the right half of the image. Part of the external carotid artery (ECA) was extracted with the ICA during segmentation since the common carotid artery bifurcates into an ICA and an ECA. The branch closest to the center of the image was identified as the ICA.

To extract the skeleton curve of an artery, we reconstructed the artery surface based on the segmentation, the whole artery was like a curved tube. We started with one end of the tube, found the center of the intersection plane, and then used this point as the source point to calculate the level curves on the surface. The centers of these level curves formed the skeleton curve representation of the artery. We extracted the curve representation for both left VA and right VA.

#### Determination of the scan positioning

To determine the scan positioning of VAs, we decided to identify the two turning points at the level of cervical vertebra 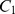 and 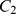 on each VA. Since the shape of the VA varies substantially, it is difficult to detect the turning points directly. We first detected a rough turning points region of VA by comparing the derivative in 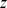 direction of the VA to a corresponding template (left VA derivative template or right VA derivative template, obtained from the averaging of ten typical subjects). The matching of the template and VA derivative was based on cross-correlation. The cross-correlation 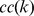 depended on the offset 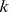 between the VA derivative of the subject and the template.

Let 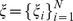 be the VA derivative of a subject and 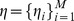 be the corresponding template. The cross-correlation between 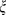 and 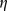 is
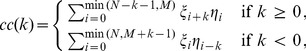
(2)where 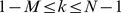. We could identify the best matching by finding 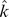 such that 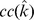 was the largest.

Using the positions of the turning points of the template, we found a rough turning points region of the subject VA. We then need to find the precise positions of the turning points of the subject VA. The rough region we got can be segmented into three subregions by the two turning points. Hence we defined the turning points as the best dividing points such that the total variance of the three subregions was minimized. More precisely, let 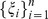 be the VA derivative in the turning point region. Let 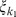 and 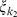 be the VA derivatives at the two turning points 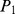 and 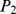. In other words, 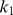 and 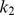 are the indices of 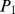 and 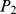. Then
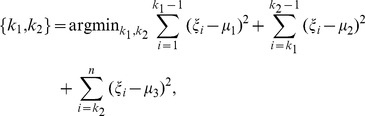
(3)where



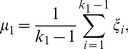
(4)

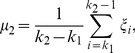
(5)




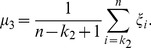
(6)


The best dividing points (or 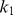 and 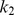) could be found by dynamic programming [18]. The middle point of the two turning point was selected to place the scan plane.

**Figure 4:**
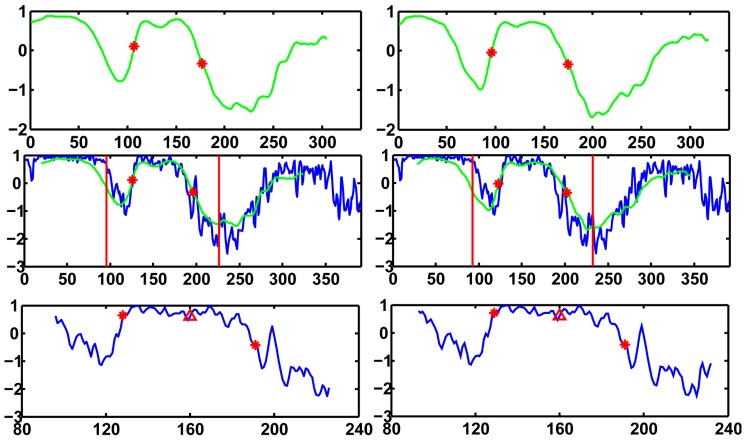
Effects of the template on turning points detection. Top panels show two left VA derivative templates and red stars show the turning points of the templates. Middle panels show the matching between the left VA derivative of a subject and the two templates. Red vertical lines show the rough regions of turning points which are determined by padding around the turning points of the templates. Bottom panels show the regions determined by matching with the templates. Red stars show the turning points detected and red triangles show the position of the scan plane.

The template was used to find a rough turning points region of the subject VA. Once the region was found, the detection of the turning points would not depend on the template. Hence the method had some tolerance about the inaccuracy of the template. However, if the template was not good enough in determining the region to work with, the method would fail. To test the effect of the template, a second template was obtained from the averaging of another ten subjects. Figure 4 shows the turning points detection results on left VA of a subject using both templates. The regions found (between the two red bars) based on template matching were different. However, the turning points detected were the same.

**Figure 5:**
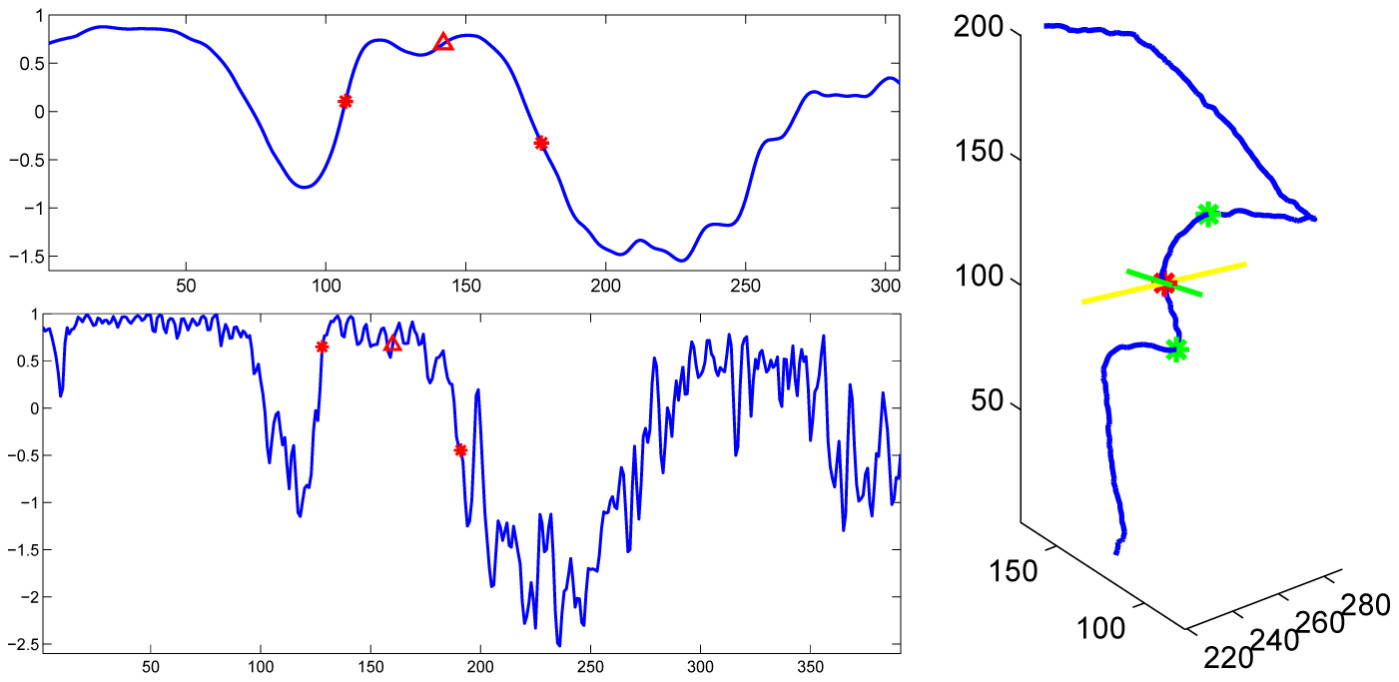
Determination of the scan positioning of the left VA. Top left panel shows the left VA derivative template, bottom left panel shows the left VA derivative of a subject. Red stars show the turning points and red triangles show the position of the scan plane. Right panel shows the positioning in 3D. The blue curve shows the left VA. The green points show the turning points. The red point and the yellow and green lines show the scan plane.

The angulation of the PC-MRI imaging slice was determined so that the normal direction of the plane was along the tangent of the artery skeleton curve, where the tangent vector was determined by the best fitting line in a small neighborhood. An example of left VA scan positioning is shown in Figure 5.

**Figure 6:**
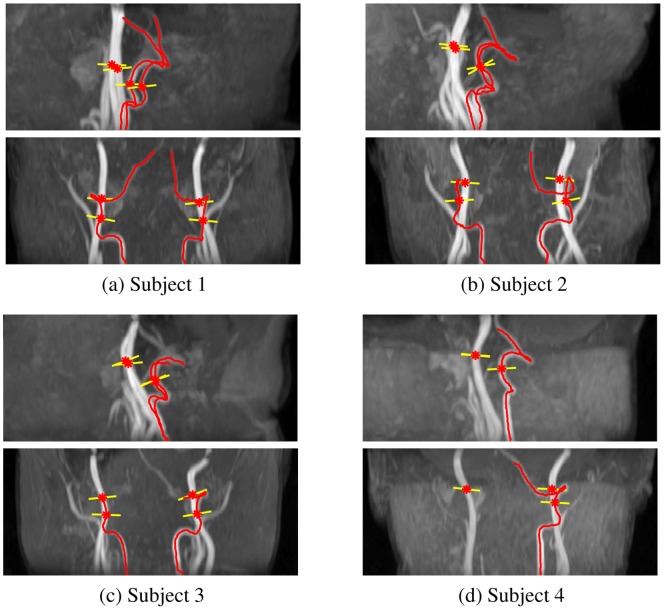
Illustration of the automatic PC-MRI scan positioning shown on 2D MIP images of the 3D axial TOF angiogram for 4 subjects. The trace of the VAs are shown in red lines and optimal PC-MRI scanning positions of the four major brain feeding arteries are shown in yellow lines, with artery centers shown as red stars.

To decide the scan positioning of the left ICA, we identified the intersection point of the left ICA and the horizontal plane passing through the upper turning point on the left VA. That point was selected to place the scan plane. The angulation was determined in the same way as that for VAs. The scan positioning of the right ICA was decided similarly. Figure 6 shows the automatic PC-MRI scan positioning results for four subjects.

### Evaluation Study

Experiments were performed on a 3-T MR system (Philips Medical Systems, Best, The Netherlands) using body coil transmission and head coil reception. The study protocol was approved by the Institutional Review Board of the University of Texas Southwestern Medical Center, the University of Texas at Dallas and the VA North Texas Health Care System. All subjects gave informed written consent before participation.

An axial 3D TOF angiogram was used to visualize the feeding arteries of the brain and to provide input to the automatic positioning algorithm for PC-MRI slices. The top of the angiogram imaging slab was positioned at the level of the bottom of pons, with the bottom slice at the level of 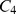 vertebra. This allowed the operator to visualize the feeding arteries of the brain which is necessary for PC-MRI slice positioning. The imaging parameters of the TOF angiogram were: 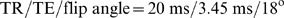, 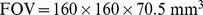, 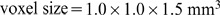, number of slices = 47, one 60 mm saturation slab positioned above the imaging slab, scan duration = 1.4 min.

The automatic positioning algorithm was implemented on a Dell workstation with a 2.4 Hz Intel xeon 6-core CPU processor (E5645) and 16G RAM. The operator exported the PAR/REC files of the TOF angiogram from the scanner console, and copied them to the workstation for processing. Once the algorithm finished the processing and reported the three off-center and three angulation parameters for each feeding artery, the operator can then input these six parameters onto the scanner console to position the PC-MRI scan of the corresponding artery.

Two studies were performed to evaluate the performance of the automatic positioning algorithm. Study 1 (N = 7) was a technical study in which the participants were recruited solely for the purpose of the present study. Accuracy and reproducibility were examined. Study 2 (N = 157) was performed to evaluate the practicality of the proposed procedures, by attaching the sequences to ongoing studies of real patient cohorts (i.e. not just highly motivated young controls).

#### Study 1: a comparative test-retest study between automatic and manual positioning

The purpose of this study has two folds: first is to evaluate the accuracy of the whole brain CBF quantification using PC-MRI with the automatic positioning by comparing to that positioned by an experienced operator; second is to evaluate the reproducibility by comparing the measurements of whole brain CBF between repeated sessions.

Seven young, healthy subjects (4 males, 3 females, 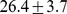 years) were examined. Each subjects had two MRI sessions continuously but with a 5 min break in the middle. The subjects came out of the scanner room after the first session and were repositioned for the second session. In each session, the subjects had the 3D TOF angiogram, four PC-MRI scans manually positioned by an experienced operator (PL), and four PC-MRI scans automatically positioned by the algorithm. Each set of the four PC-MRI scans targeted the four feeding arteries of the brain, left ICA, right ICA, left VA and right VA, respectively. The order of manual positioning and automatic positioning was counterbalanced across subjects. The operator for manual positioning was blinded from positioning information given by the automatic algorithm.

The manual positioning of PC-MRI scans was based on the 2D MIP images of the 3D axial TOF angiogram, and utilized the same criteria as those in the automatic positioning algorithm. Specifically, the PC-MRI slices for ICAs were placed at the level of foramen magnum, and slices for VAs were placed to be in the middle between the two turns of VAs at the level of cervical vertebra 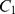 and 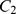. For all PC-MRI scans, the center of the FOV was placed to overlap with the center of the targeted artery, and the imaging slices were tilted to be perpendicular to the targeted artery. Imaging parameters of PC-MRI are: single slice, 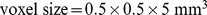, 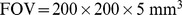, maximum velocity encoding = 80 cm/s, 4 averages, scan duration of one PC-MRI scan is 0.5 min.

In addition, a 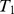-weighted magnetization-prepared rapid gradient-echo (MPRAGE) image (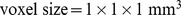) was acquired in the first session of each subject to provide an estimation of the brain volume for the quantification of CBF in the unit of ml/100 g tissue/min.

Data processing of the PC-MRI followed methods used previously [10], [11]. Briefly, a ROI was manually drawn on the targeted artery of each PC-MRI scans based on the magnitude image. The operator was instructed to trace the boundary of the targeted artery without including adjacent vessels. The phase signals, i.e. velocity values, within the mask were summed to yield the blood flux (in ml/min) of each artery. The whole brain CBF (in ml/100 g/min) was further obtained by normalizing the total flux of all four arteries to the intracranial mass (in gram), which was estimated from the high resolution 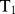-MPRAGE image using the software FSL (FMRIB Software Library, Oxford University).

For each PC-MRI positioning strategy and for each artery, the reproducibility index, inter-session Coefficient of Variation (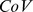), was calculated as follows:
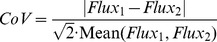
(7)where 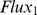 and 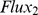 represent the measurement #1 and #2 from the two sessions, respectively. Note that the inter-session 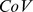 is expected to contain repositioning error, PC-MRI measurement noise, and subject physiologic variation.

Since the center of the FOV of the PC-MRI scans was attempted to be placed to overlap with the center of the targeted artery in both manual and automatic positioning, the distance between the center of the targeted artery on the resulting PC-MRI images and the center of the image was calculated. The size of the targeted artery on the resulting PC-MRI images was also recorded based on the manual boundary tracing.

The agreements between the blood flow values measured by PC-MRI with automatic positioning and manual positioning were evaluated with the Bland-Altman method [19], [20]. Bland-Altman method was also used to compare the measurements between the two sessions. Two-way ANOVA with repeated measures was performed to compare between the four feeding arteries and between the two positioning strategies using the averaged blood flux of the two sessions. The same analyses were performed for inter-session CoV, artery sizes, and off-center distance of the targeted artery on PC MRI imaging slice. In all analyses, a 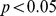 is considered statistically significant.

#### Study 2: a large-scale application study

For study 2, a total of 157 subjects (83 males and 74 females) ranging from 13–55 years old were studied, including 71 patients and 86 healthy controls. Among the 71 patients, 19 have schizophrenia, 18 have anorexia, 18 have marijuana dependence and 16 have cocaine dependence. Thus, a total of 628 (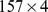) arteries were evaluated to test whether it is feasible to utilize our algorithm on a routine basis. Each subject had the identical 3D axial TOF angiogram as described earlier. The resulting axial images were input to the automatic positioning algorithm for processing. The failure cases, including the cases in which the algorithm failed to provide the six positioning parameters as well as the cases in which the algorithm output were not correctly targeting the corresponding arteries were recorded and carefully investigated.

Based on whether the algorithm was successfully providing the positioning information, the 157 subjects were grouped into three subsets: all successful group (successfully positioned all four feeding arteries), one-artery failed group (successfully positioned three of the four feeding arteries), and all failed group (failed to position all four arteries, the algorithm would not return positioning information if less than three arteries were detected.). Given that arterial size and amount of motion (thereby SNR of the TOF images) may be dependent on subject age, gender and subject category (control or patient), the PC-MRI data were evaluated by the Chi-square test of independence on the contingency tables. The subjects are grouped into three age groups for this analysis: 13–24 years old (N = 47), 25–40 years old (N = 55) and 41–55 years old (N = 55). A 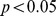 is considered statistically significant.

## Results

### Study 1

For study 1, all seven subjects successfully completed the two sessions, and the automatic positioning algorithm successfully provided positioning information for all arteries. So a total of 28 arteries were measured and each artery was measured twice by the automatic positioning method and twice by the manual one.

**Figure 7:**
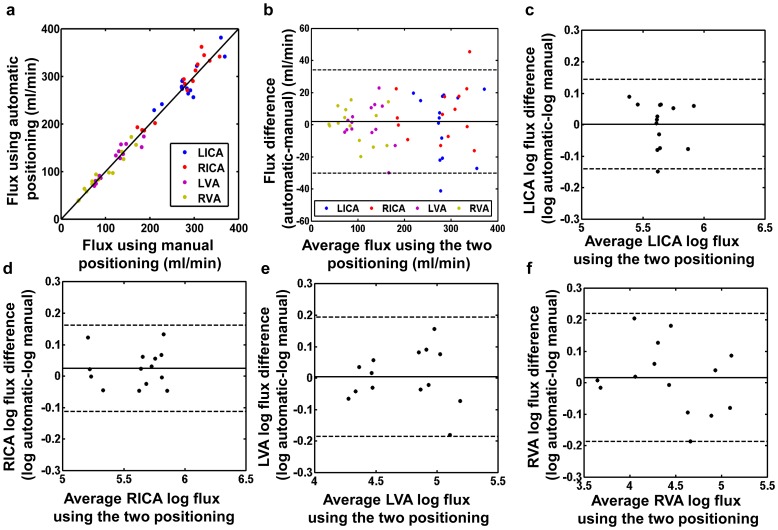
Comparison between the blood flow measurements using the automatic positioning algorithm and using manual positioning. (a) Scatter plot of the two blood flow measurements: automatic versus manual. Each color represents the data points from one kind of artery. Each dot represents data from one artery of one subject in one session (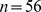). The solid line indicates the line of equality.(b) Scatter plot of the two blood flow measurements: difference (automatic-manual) versus average of values measured by the two positioning methods. The solid line indicates the mean difference between two measurements. (c)-(f) Bland-Altman plot comparing the blood flow measurements of each of the four arteries (LICA, RICA, LVA and RVA) obtained by the two positioning methods after 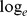 transformation (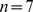, with 2 replicates for each artery of each subject). For all the Bland-Altman plots, the solid line indicates the mean difference between two measurements. The dashed lines indicate the 95% confidence interval.

**Figure 8:**
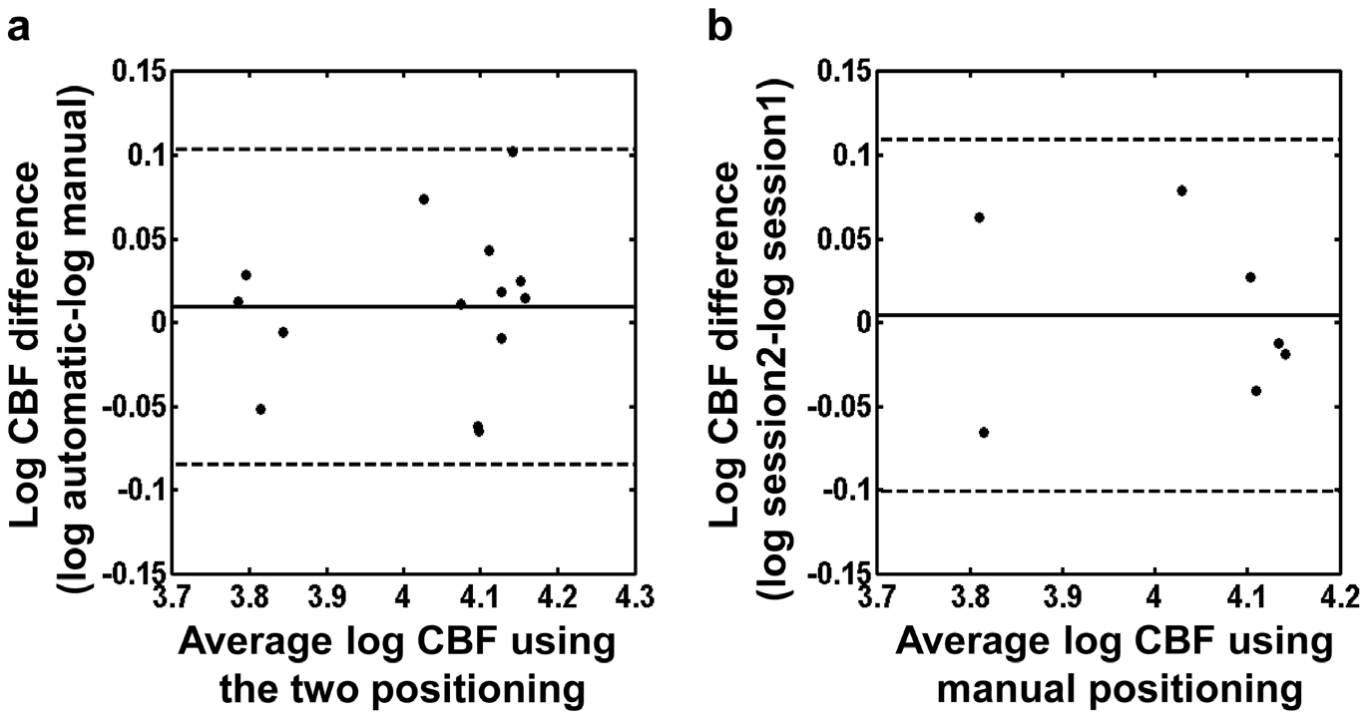
Comparison between the whole brain blood flow measurements using the automatic positioning algorithm and using manual positioning. (a) Bland-Altman plot comparing the whole brain cerebral blood flow measurements obtained by the two positioning methods after 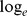 transformation (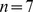, with 2 replicates for each subject). (b) Bland-Altman plot comparing two whole brain cerebral blood flow measurements obtained by the manual positioning method after 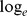 transformation (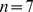). For all the Bland-Altman plots, the solid line indicates the mean difference between two measurements. The dashed lines indicate the 95% confidence interval.

A two-way ANOVA with repeated measures of arteries (LICA, RICA, LVA, RVA) and positioning methods (automatic, manual) on the quantified average flux values of the two sessions was conducted. The main effect of the positioning strategy was not significant (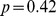). Thus, the automatic positioning algorithm does not create systematic bias in the results, as compared to an experienced operator. Figure 7a shows a scatter plot of the flux measurements using the two positioning strategies: automatic versus manual. Figure 7b shows a scatter plot of the flux measurements: difference (automatic-manual) versus average of values measured by the two positioning methods. Figure 7b shows that the difference between measurements increases as the flux measurement rises. Hence the logarithmic transformation was applied to the measurements to remove this relationship. Figures 7c–f show the Bland-Altman plots using 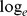 transformed flux measurements for each of the four arteries (LICA, RICA, LVA and RVA). The geometric mean ratios of LICA, RICA, LVA and RVA flux values by the automatic and manual methods are 1.002, 1.025, 1.004 and 1.017 respectively. The automatic method differs from the manual method by 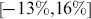, 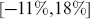, 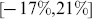 and 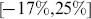, for 95% of LICA, RICA, LVA and RVA flux measurements respectively. Bland-Altman method was also applied to the whole brain CBF measurements using the two positioning strategies (Figure 8). The geometric mean ratio of CBF values by the automatic and manual methods was 1.01. The automatic method differs from the manual method by between 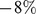 and 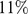, for 95% of whole brain CBF measurements. This is comparable to the variance in CBF measurement using manually-positioned PC MRI alone, which is between 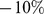 and 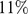. Previous studies have reported that the CBF variance using Positron Emission Tomography (PET) is about 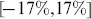 [21], and that using ASL technique is about 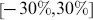 [22]. Therefore, the variance between automatic and manual positioning strategies we observed in CBF measurements is within the limit of current CBF technologies. The mean whole brain CBF across subjects were 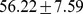 ml/100 g/min and 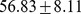 ml/100 g/min from the automatic positioning and manual positioning of the PC-MRI scans, respectively. A paired t test between results using the two positioning methods (automatic vs. manual) was conducted using the mean CBF values of the two sessions. The effect of the positioning strategy was not significant (p = 0.42).

The inter-session 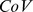 for the flux measured with automatic PC-MRI positioning was 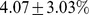 across all 28 arteries, and that measured with manual positioning by an experience operator was 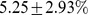. A two-way ANOVA with repeated measures of arteries (LICA, RICA, LVA, RVA) and positioning methods (automatic, manual) on inter-session 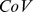 was conducted. The main effect of positioning strategy was not significant (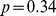). It can be seen that the inter-session 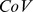, which includes the repositioning noise, the measurement noise, and subject physiologic fluctuations, is considered low for the automatic positioning, suggesting high reliability of the automatic positioning algorithm used.

Location of the target artery relative to the center of the PC-MRI image was examined. The off-center distance was 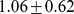 mm when using the automatic positioning algorithm and 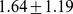 mm when using manual positioning. A two-way ANOVA with repeated measures of arteries (LICA, RICA, LVA, RVA) and positioning methods (automatic, manual) on mean off-center distance of the two sessions was conducted. The positioning method has a trend of significant effect with p = 0.089. A two-way ANOVA with repeated measures of arteries (LICA, RICA, LVA, RVA) and positioning methods (automatic, manual) was also conducted on the estimated size (in 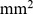) of the arteries from the acquired PC-MRI images. The main effect of the positioning strategy was not significant (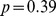).

### Study 2

For study 2, the automatic positioning algorithm was applied to a separate group of 157 subjects. The average processing time of the algorithm was 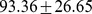 seconds, ranging from 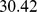 seconds to 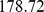 seconds.

As shown in Table 1, in 125 subjects, the algorithm successfully provided information on all four arteries with a subject-based successful rate of 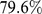. For the rest, the algorithm failed to provide any information in 11 subjects. In another 20 subjects, the algorithm successfully provided positioning information for three arteries but one. There was one subject for whom the algorithm provided wrong positioning information for one artery. Therefore, out of a maximum of 628 (i.e. 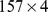) arteries, the algorithm successfully provided the positioning information for 563 arteries, equivalent to a success rate of 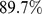. Since the operator had to position PC scans on the failed arteries manually, this indicates that the operator’s work load was reduced by 89.7%.

**Table 1:** Subject information in groups and the p values of the Chi-square tests of independence.

		Performance Groups			Chi-square test of Independence
		Allsuccessful	One-arteryfailed	Allfailed	*p* value
Total number of subjects	157	125	21	11	–
Age groups	Age 13–24	38	5	4	0.32
	Age 25–40	47	7	1	
	Age 41–55	40	9	6	
Gender	Male	66	12	5	0.82
	Female	59	9	6	
Category	Control	71	10	3	0.14
	Patient	54	11	8	

Further investigation showed that, the 11 subjects, for whom the automatic positioning algorithm failed for all four arteries, can be separated into two types: 1) the quality of angiogram was bad due to subject motion (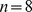). As a result, the algorithm failed at the segmentation step due to low contrast to noise ratio. 2) both the left VA and right VA are small in the subjects, consequently the algorithm aborted as designed when it failed to identify at least three arteries (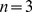). In the 20 subjects for whom the algorithm only provided positioning information for three arteries, the one artery that failed to be identified always appeared thin in the angiogram, and it was always one of the VAs which typically has smaller size than the ICAs. For the one artery that the algorithm provided wrong information, it was because the algorithm falsely identified the right ECA as right ICA.

We would like to know if the age, gender and subject category (control/patient) affect the algorithm performance. Since the algorithm performance values in this study take ordered discrete values (0, all successful, 1, one-artery failed, 2, all failed), analysis-of-variance (ANOVA) can not be applied. We use the Chi-square test of independence on the contingency table. The subjects were grouped into three age groups (13–24 years old,15–40 years old,41–55 years old). The Chi-square test showed no significant effect of age (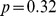) and gender (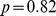) (Table 1). There were more patients than control subjects in the all failed group, but the effect of subject category was not significant (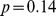).

## Discussion

The present study proposed a novel automatic algorithm for the positioning of PC-MRI at the feeding arteries of the brain. The algorithm takes a 3D TOF angiogram dataset as input, and generates six positioning parameters for each of the feeding artery as output, which can then be directly typed in the MRI user interface to define the imaging slice location and angulation of the PC-MRI scans. The comparative test-retest study showed that the PC-MRI measured blood flow using the automatic positioning algorithm is precise (inter-session 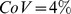) and consistent with that measured with manual positioning by an experienced operator (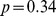). Further application of this algorithm in 157 consecutive participants demonstrated that it is feasible to utilize the proposed procedure in typical clinical populations.

PC-MRI, as a noninvasive technique to measure blood flow accurately and rapidly, has been commonly used in clinic and research to quantify whole brain CBF [7]–[9]. It has also been utilized to normalize other regional CBF methods such as ASL and DSC-MRI [5], [6], [10]. Some studies used a single PC-MRI slice at the level of cervical vertebrae 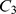 to measure the brain’s four feeding arteries, where all four arteries (left/right ICA, left/right VA) were parallel to each other [5], [6]. While this position allows the arteries to be “captured” in one PC scan, it could introduce bias caused by complications such as arterial branching outside of brain, lower gradient strength as away from iso-center of the scanner, and late branching of common carotid artery which would result in the inclusion of blood from external carotid artery. Therefore, it has been suggested to acquire PC-MRI at a higher location immediately adjacent to foramen magnum using four separate scans in order to achieve more accurate quantification of whole brain CBF [11]. However, such positioning of PC-MRI is not trivial due to large variability in arterial trajectory across individuals. There has been no available tool to assist the slice positioning of PC-MRI scans, thus considerable training and experience are needed for the operators. Our automatic positioning algorithm was designed to mitigate this problem by making the PC-MRI measured CBF minimal operator dependent.

Our results showed that, the inter-session 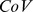 of blood flow measurement using the proposed automatic positioning algorithm was comparable to, if not better than that obtained with manual positioning by an experienced operator. The resulting blood flow quantification was also consistent between the automatic and manual positioning (Figures 7, 8). We note that the increase of difference between the two positioning methods at larger blood flow is due to the increase of physiologic noise associated with larger blood flow. These results suggested that our PC-MRI automatic positioning algorithm could achieve reliable blood flow measurements without special needs in the operators’ skills and experience. This feature would make this algorithm optimal for test-retest studies and multi-center studies, as the operator-related variation could be minimized.

The efficacy of the proposed automatic positioning algorithm was evaluated in a large scale study with 157 subjects. The subjects population includes wide age range, both gender, and both healthy controls and patients. The patients included had no known vascular diseases. The algorithm takes an average of 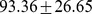 seconds to execute, with an artery-based success rate of about 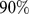. The failure of the algorithm is mainly caused by the vertebral arteries being too small (on one side or on both sides), and the quality of 3D TOF angiogram being poor due to subject’s motion. Although the subject’s age and gender might affect the quality of the TOF angiogram due to their effect on blood flow velocity [23], [24], the results of the large scale study suggested that they have no apparent effect on the performance of the algorithm. However, the patients (with either schizophrenia, drug addiction, or anorexia) tend to have more motions that made the algorithm completely fail for all arteries although the effect is not significant (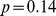). Although further improvement is needed, these results showed that the proposed automatic positioning algorithm could be utilized in a broad range of studies.

Multilevel threshold based segmentation method is used to identify the four brain feeding arteries because this kind of method is usually fast, and the automatic positioning algorithm needs to be fast enough to be used during a scan session. The successful rate of the automatic positional algorithm heavily depends on the segmentation step. To improve the successful rate, further investigation of fast artery segmentation method is needed, especially in the cases of thin vertebral arteries and apparent subject motions. Another limitation of the current algorithm is the requirement of a minimum of three detectable arteries in order for the algorithm to generate the positioning outputs. This requirement was placed in the algorithm because at least one VA is needed to determine the z-coordinate of the foramen magnum. However, this constraint precluded the algorithm from being applied to individuals in whom both sides of the vertebral arteries are small in caliber.

A practical limitation is that, at present, the algorithm script is being performed on a dedicated workstation (different from the scanner console). As a result, the workflow of the procedure is that the operator needs to export the 3D TOF data and transport them to the workstation for processing, and then type in the algorithm output back into the scanner console. We are working with our MRI vendor to implement the algorithm on the scanner console and the output of the algorithm will be directly imported to the scanning software, which will further improve the practicality and workflow of the proposed procedure.

## Conclusions

We have proposed an automatic positioning algorithm for the placement of PC-MRI scans in the estimation of global CBF. The algorithm is shown to be capable of producing precise and accurate scanning guidance compared to manual positioning by an experienced operator. Further application of the algorithm demonstrated the feasibility of utilizing this procedure in typical clinical populations. Although further improvement is needed, this automatic positioning algorithm would improve the application of PC-MRI in CBF quantification.
